# Effects of GUASHA on Heart Rate Variability in Healthy Male Volunteers under Normal Condition and Weightlifters after Weightlifting Training Sessions

**DOI:** 10.1155/2015/268471

**Published:** 2015-06-10

**Authors:** Xingze Wang, Uraiwan Chatchawan, Saowanee Nakmareong, Atit Silsirivanit, Yingying Wang, Dongbei Xie, Jinsheng Yang, Wichai Eungpinichpong

**Affiliations:** ^1^Research Center in Back, Neck, Other Joint Pain and Human Performance, Khon Kaen University, Khon Kaen 40002, Thailand; ^2^Faculty of Physical Education, Gannan Normal University, Ganzhou 341000, China; ^3^Research and Training Center for Enhancing Quality of Life of Working-Age People, Khon Kaen University, Khon Kaen 40002, Thailand; ^4^Faculty of Associated Medical Sciences, Khon Kaen University, Khon Kaen 40002, Thailand; ^5^Faculty of Medicine, Khon Kaen University, Khon Kaen 40002, Thailand; ^6^Institute of Acupuncture and Moxibustion, China Academy of Chinese Medical Sciences, Beijing 100700, China

## Abstract

*Objectives*. This paper aims at exploring the effects of GUASHA on heart rate variability between healthy volunteers under normal condition and weightlifters after training sessions.* Methods*. Ten healthy male volunteers under normal condition and 15 male weightlifters after weightlifting training sessions were recruited into two groups. Electrocardiography was recorded before and immediately after 20-minute GUASHA. HRV was calculated in both the time domain and the frequency domain.* Results*. Stress index was reduced, while standard deviation of N-N intervals (SDNN), proportion derived by dividing the number of interval differences of successive N-N intervals greater than 50 ms, and root mean square of successive differences (RMSSD) were enhanced after GUASHA therapy in the two groups. The changes in SDNN and RMSSD were higher in the healthy men group than in the weightlifters group. In addition, low frequency was decreased whereas high frequency was significantly increased in healthy men after the GUASHA session.* Conclusions*. GUASHA therapy facilitates the parasympathetic nervous activity and modulates the balance between parasympathetic and sympathetic activities in both healthy men under normal condition and weightlifters after training sessions as indicated. Although the changes of the HRV parameters were similar in both groups, the responsiveness was more pronounced in healthy men than in male weightlifters.

## 1. Introduction

GUASHA is a traditional Chinese therapy in which skin is scraped repeatedly by using a blunt spoon-like object on certain areas of the body. It is performed along the meridians with different kinds of tools and manipulation techniques to cause petechiae and ecchymosis for treatment and health care. “GUA” means to scrape or rub; “SHA” means petechiae and ecchymosis as well as the feeling of satisfaction. The physiological mechanism of GUASHA in both healthy people and patients is that it increases surface microperfusion [1]. GUASHA has an immune anti-inflammatory effect due to upregulating the heme oxygenase-1 [2], prolonging the endurance time [3], enhancing the amount of white blood cells and neutrophils [4], and reducing muscle pain and chronic fatigue syndrome [5, 6].

Heart rate variability (HRV) is used as a marker of fatigue [7]. HRV is the tiny variation of beat-to-beat of the heart. It refers to various computed outcomes used to quantify the time intervals between heart beats as well as beat-to-beat changes in one single interval. Physiological studies show that high variability of the interbeat interval generally corresponds to the enhanced parasympathetic nervous activity whereas low variability of the interbeat interval generally corresponds to the strengthened sympathetic nervous activity [8]. HRV can be changed by acupuncture therapy [9–11]. Some of the other therapies such as moxibustion, tuina, cupping, and GUASHA [12] have similar effects with acupuncture. However, few studies have reported the effects of GUASHA therapy on HRV. We hypothesized that GUASHA can reduce the exercise fatigue of the athletes, such as weightlifters. Objective of the present study was to investigate the possibility that HRV can be used to detect changes in autonomic nervous activity after GUASHA therapy in healthy male volunteers under normal condition and male weightlifters after weightlifting training sessions.

## 2. Materials and Design

The same manipulation was conducted to test HRV during a session in healthy male volunteers and male weightlifters by a research group including one GUASHA therapist, a neurologist, and a research methodologist/statistician. The ethic committee of Khon Kaen University approved the research protocol. Written informed consent was obtained from all the participants before the study. This study was conducted in the Division of rehabilitation, Faculty of Physical Education, Gannan Normal University, China.

### 2.1. Environment and Procedure

**Figure 1:**
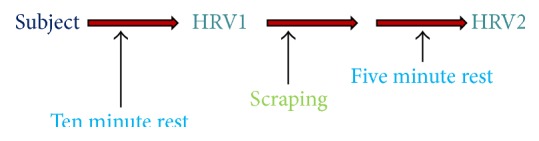
Trial flowchart.

Each of the participants underwent a process of HRV measurements and scraping as shown in Figure 1. Measurements were made in a quiet room with about 50% humidity and average temperature of 27 ± 1°C, without direct sunlight, without infrared radiation, and without indoor-outdoor ventilation. Before measurements, the subjects were instructed to rest more than 10 minutes in this quiet and relaxing atmosphere. Subjects were further instructed to be in a sitting position wearing an ear sensor and to breathe at a frequency of 1 breath/4 s (0.25 Hz) in synchrony with the sound of an electric metronome at comfortable volume levels.

### 2.2. Subjects

All subjects were college students or active athletes who underwent a physical examination to determine that they had no health problems or injuries. The inclusion criteria were no smoking, no alcohol, and no history of hormone therapy. Exclusion criteria were with joint or muscle dysfunction, smoking, or drinking alcohol. Data were collected in China from 25 male volunteers, in whom there were 10 university students and 15 weightlifters. The baseline features were as follows: the average age was 21.6 ± 3.4 years, the average weight was 54.2 ± 31.8 kg, the average height was 172.8 ± 8.1 cm, and the average body mass index was 24.6 ± 4.2. For the male students, they have never joined professional training. Futhermore, their exercises were aimed to improve health fitness, such as, exercises were performed 3 sessions per week, more than 30 minutes per session, and reach 85% of maximum heart rate. For the male weightlifters, the average duration of training experiences was 4.2 ± 0.7 years, the average active athletic level was 2.0 ± 0.5 (note that the titles of athletes in China included international masters of sports (1++), masters of sports (1+), first grade (1), second grade (2), third grade (3), and young athletes (4)). Definition of normal condition and weightlifting training sessions is as follows: (a) normal life in healthy male university student involves having classes in the morning and afternoon and doing exercises on Monday, Wednesday, and Friday afternoon with middle intensity load for half an hour; (b) normal weightlifting training sessions in male weightlifters involve training from Monday to Saturday, on Wednesday with 85% 1RM, on Friday with 90% 1RM, and on Saturday 90–95% 1RM on snatch or clean and jerk. All the participants were required to get up before 8:00 am on the day of experiment. The subjects live with the same lifestyle based on the previous life before and during the experiment day.

*Recording of HRV*. HRV was measured on Monday morning in that all the volunteers were in equal and basal physical conditions. Data was recorded (version 4.2, biofeedback 2000 x-pert software, made in Austria) from the chest wall for 5 minutes before and after GUASHA. The sampling frequency was 100 Hz. The red electrode cable was attached to the left chest in the area of the fifth rib and the blue one to the right chest; the reference can be attached to the Adam's apple. Various temporal indications can be calculated including stress index (SI), standard deviation of N-N intervals (SDNN), proportion derived by dividing the number of interval differences of successive N-N intervals greater than 50 ms (pNN50), root mean square of successive differences (RMSSD), low frequency (LF), high frequency (HF), and the ratio of LF/HF which is a direct index of HRV.

### 2.3. GUASHA Intervention

The tool of GUASHA was the Buffalo Horn scraper and a skin lubricant to decrease friction. The therapist has been trained and certified by the Ministry of Human Resources and Social Security, China. The head, neck, and back were scraped with 45 degrees between the scraper and skin in the direction from up to down and center to edge. The number of times of GUASHA: head (60), neck (60), back (whole 40, up 40, middle 40, low 20). Total GUASHA time was 20 minutes and it took place between 9:00–11:30 am. Pressure of GUASHA by the GUASHA therapists was mild (487 ± 21.4 g) and moderate (626 ± 10.6 g), based on the measurements using AlgoMed System. “SHA” describes stasis within the tissue as well as the petechiae raised from GUASHA signifying liberation of that stasis [13].

### 2.4. Data Analysis

Data were presented as mean ± standard deviation (SD). Within group, paired sample* t* test was used to compare the results, and independent sample* t* test was used to analyze the difference between groups. Statistical analysis was performed using SPSS 17.0 Software. To achieve the statistical significant, 80% power and an alpha level of 0.05 were used.

## 3. Results

### 3.1. The Effects of GUASHA Therapy on Time Domain

**Figure 2:**
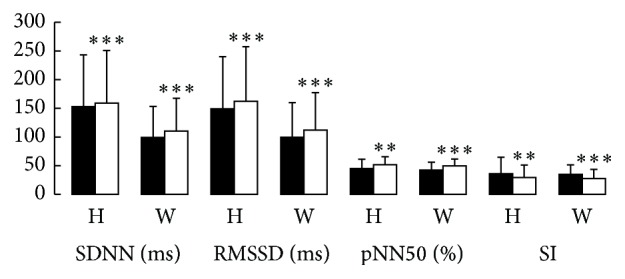
The changes in the time domain of HRV before and after GUASHA in the healthy males under normal condition and weightlifters under weightlifting training condition. The black bar means before GUASHA. The white bar means after GUASHA treatment. H means healthy males; W means male weightlifters. SDNN means standard deviation of N-N intervals; SI means stress index; pNN50 means proportion derived by dividing the number of interval differences of successive N-N intervals greater than 50 ms; RMSSD means root means square of successive differences. The units in *y*-axis are different in accordance with units in the brackets of the *x*-axis items, ^**^ *P* < 0.01. ^***^ *P* < 0.001 means the time domain of the subjects before GUASHA versus after GUASHA.

The changes in time domain of HRV include SI, SDNN, RMSSD, and pNN50.  Table 1 showed the values before and after GUASHA therapy. The results revealed that the SI was significantly reduced, while SDNN, RMSSD, and pNN50 were improved in both groups. When the two groups were compared, SDDN and RMSSD were higher in the healthy group while pNN50 and SI kept the same trend during the GUASHA therapy, Figure 2.

### 3.2. The Effects of GUASHA Therapy on Frequency Domain

**Figure 3:**
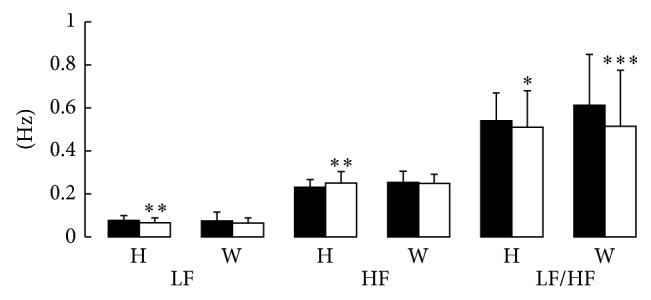
The changes in the frequency domain of HRV before and after GUASHA in healthy males under normal condition and male weightlifters under weightlifting training condition. LF and HF significantly improved in the healthy males group, while LF/HF were significantly reduced in both groups. The black bar means the value before GUASHA treatment. The white bar means the value after GUASHA treatment. H refers to healthy males; W means male weightlifters; LF is short for low frequency; HF is short for high frequency. ^*^ *P* < 0.05. ^**^ *P* < 0.01. ^***^ *P* < 0.001 versus before treatment.

Figure 3 shows the changes in frequency domain of HRV. These values were significantly different before and after GUASHA (*P* < 0.05). These values were induced due to GUASHA therapy, Table 2. It revealed that LF and HF significantly improved in the healthy males group, while LF/HF were significantly reduced in both groups. When comparing the two groups, HF was higher in the healthy group than in the weightlifters group for baseline level (*P* < 0.01, independent* t* test), whereas LF, LF/HF kept the same trend during the GUASHA therapy in the two groups.

## 4. Discussions

### 4.1. The Effects of GUASHA Therapy on Time Domain

The recording of HRV in healthy people was used to modulate and monitor health care in previous studies [14–17]. Time domain including SI, SDNN, RMSSD, and PNN50 analyzes the changes in heart rate over time or intervals between successive normal cardiac cycles [18]. SI responds sensitively to changes in the vegetative balance between the sympathetic and parasympathetic nervous system. SDNN describes the total regulation of the autonomic nervous system to cardiac function. RMSSD indicates how much the heart rate varies from one heart beat to the next. pNN50 is an indication of parasympathetic activity.

Farinatti et al. studied that the change of time domain, including SDNN, RMSSD, and pNN50, increased after stretch session such that their values enhanced after the stretch session [19]. Another research demonstrated the same trend after mild physical exercise on change of time domain [20], whereas our study also demonstrated the same results which were that the SDNN, pNN50, and RMSSD enhanced significantly after GUASHA treatment. Nevertheless, there was a difference between both groups in time domain such that the values of SDNN and RMSSD in healthy group were more than those in weightlifters in our study. The reason may be different parasympathetic nervous activities due to the weightlifters having been trained for 4 years in this study. The effect of habitual weightlifting training influences baseline parasympathetic tone in HRV. Furthermore, the value of the weightlifters was lower than that of the healthy males, as players' physical fatigue was higher than that of average people. The previous studies showed some difference in HRV between the healthy group and the weightlifters [21, 22].

Another study demonstrated that the effects of exercise training on HRV which enhanced in HRV due to appropriate exercise on health promotion, such as, increasing the SDNN and RMSSD after exercise and training [23]. RMSSD showed an increase after GUASHA therapy, expressing the strengthened activity of the parasympathetic function by GUASHA in this study; however, the difference between healthy males and players may be in that RMSSD was not in the same active level in the parasympathetic nervous system between these two groups. SI was lower in the two groups after GUASHA. The reasons of the same changes may be in that the weightlifters were also healthy people. Also, the same meridians were used in both acupuncture and GUASHA therapies [12].

The GUASHA therapy is beneficial for health care and treatment because it effectively improves microcirculation [1] and skin temperature [24], and it upregulates heme oxygenase-1 [2] and reduces muscle pain and chronic fatigue syndrome [5, 6]. The previous study demonstrated that GUASHA could reduce fatigue [4, 6]. The cutaneous regions have a defensive function and are an external indicator of the functional status of zang-fu organs in TCM [25], and skin can metabolize hormones and produce derivatives with a potentially systemic activity based on modern medicine [26–31]. The change of time domain indicated that some meridians may be stimulated in cutaneous regions and perhaps the biochemical, neurological, and endocrine changes were induced by physical therapy (acupuncture, moxibustion, GUASHA, cupping, and tuina).

### 4.2. The Effects of GUASHA Therapy on Frequency Domain

Frequency domain analysis including HF, LF, and the ratio of LF/HF describes the periodic oscillations of the heart rate signal decomposed at different frequencies and provides information on the amount of relative intensity in the heart's sinus rhythm [32]. LF represents parasympathetic and sympathetic influences, and fluctuations in this band are periodic oscillations of the arterial blood pressure as a result of rhythmic contraction of blood vessels that offer resistance to blood flow. HF band reflects how the heart rate adapts to the respiration rhythm. It is an important factor in parasympathetic activity. The ratio of LF/HF is regarded as the proportion of sympathetic and parasympathetic activity.

Chalencon et al. demonstrated the relevance of frequency domain change in HRV as a valuable tool to assess the physiological training-induced responses and to optimize athletic performance; moreover, using performance or HF as the systems' output is as the systems output provided the same information on the fatigue status of the athlete [33]. Chen et al. demonstrated that parasympathetic power indicated by HF in HRV can reflect the recovery status of the weightlifter after training, such that the HF value declined due to weightlifting training [34]. Vigorous training can suppress parasympathetic power by lowering increasing HF, whereas sympathetic power is increased by LF in HRV. So, HF power is contributed to parasympathetic nervous activity. However, HF was enhanced to display the high level of parasympathetic activity after GUASHA only in the healthy males, possibly indicating that the players belong to a special group of people whose range is limited. It was found that the effect of GUASHA therapy is beneficial for healthy people relating to parasympathetic activity function.

Our study shows that LF declined significantly after GUASHA therapy in the healthy males; however, LF has a trend of decline in weightlifters. These indicate the higher level balance between the parasympathetic and sympathetic nervous systems. LF power in normalized unit is considered as a marker of sympathetic nervous activity. Another study demonstrated that LF in HRV enhanced after weightlifting training [34]. Previous study showed that the ratio of LF/HF enhanced due to weightlifting training [21]. Increased HRV has been found when a decrease in LF/HF ratio occurs and is indicative of increased parasympathetic activity. Our study showed that LF/HF ratio declined significantly in both groups. As a result, the ratio of LF/HF declined, expressing that the parasympathetic nervous system was activated due to the GUASHA therapy in both groups.

Many mechanisms by which GUASHA may affect the skin and homeostasis have been suggested. A study on effect of mechanical pressure on the skin demonstrate that the pressure could activate fibroblastic proliferation depended on the amount and form of the applied pressure [35]. However, the “SHA” resulting from the repeated mechanical pressure (with stroke) was thought to be extravasation of blood from the peripheral capillaries [36]. These biological responses may be induced by leakage [37]. Furthermore, the GUASHA could regulate the endocrine and increase HO-1 [2, 38], releasing corticotropin hormone due to the cutaneous response to the stress [39]. The surface of the body offers evidence of internal dysfunction, and it is possible to influence the interior by the application of reflexively powerful stimuli from the skin surface. Koizumi found that stimulation of the skin in the abdomen produced profound inhibition of intestinal movement [40]. This phenomenon showed that the internal organs cannot react directly to painful stimulus, instead by producing spasm and paraesthesia in the reflexively related muscle wall, often augmented by hyperaesthesia of the overlying skin. Autonomic nervous system was stimulated due to GUASHA in cutaneous regions [41].

## 5. Conclusions

GUASHA therapy could facilitate the parasympathetic nervous activity and modulate the balance between parasympathetic and sympathetic activities in both the healthy men and the weightlifters as indicated by the changes in HRV parameters. Although the changes of the HRV parameters were similar in both groups, the responsiveness was more obvious in the healthy males than in the weightlifters.

## Figures and Tables

**Table 1:** Changes of time domain before and after GUASHA therapy.

Item	Type	Before	After
SI	Healthy (*n* = 10)	36.03 ± 28.81	29.31 ± 21.57
	Weightlifters (*n* = 15)	35.06 ± 16.41	27.57 ± 15.78
SDNN (ms)	Healthy (*n* = 10)	152.82 ± 90.37	159.05 ± 91.72
	Weightlifters (*n* = 15)	99.46 ± 53.75	110.32 ± 56.96
RMSSD (ms)	Healthy (*n* = 10)	149.37 ± 90.59	162.03 ± 95.54
	Weightlifters (*n* = 15)	99.61 ± 60.14	112.22 ± 65.13
pNN50 (%)	Healthy (*n* = 10)	45.011 ± 16.03	51.71 ± 13.86
	Weightlifters (*n* = 15)	42.51 ± 13.46	49.72 ± 11.71

**Table 2:** Changes of frequency domain before and after GUASHA therapy.

Item	Type	Before	After
LF (Hz)	Healthy (*n* = 10)	0.074 ± 0.041 Hz	0.064 ± 0.024 Hz
	Weightlifters (*n* = 15)	0.076 ± 0.023 Hz	0.065 ± 0.023 Hz
HF (Hz)	Healthy (*n* = 10)	0.23 ± 0.036 Hz	0.25 ± 0.053 Hz
	Weightlifters (*n* = 15)	0.253 ± 0.052 Hz	0.249 ± 0.042 Hz
LF/HF	Healthy (*n* = 10)	0.54 ± 0.13	0.51 ± 0.17
	Weightlifters (*n* = 15)	0.612 ± 0.237	0.515 ± 0.26
